# Bellerophon: An Automated Tool for PROTAC Decomposition

**DOI:** 10.1021/acsmedchemlett.5c00769

**Published:** 2026-04-10

**Authors:** Giulia Apprato, Matteo Bertola, Amelia Locatelli, Giulia Caron, Andrea Mauri, Giuseppe Ermondi

**Affiliations:** † Department of Molecular Biotechnology and Health Sciences, 9314 University of Turin, Torino 10126, Italy; ‡ Alvascience Srl, Lecco 23900, Italy

**Keywords:** TPD, PROTAC, decomposition tool, drug discovery

## Abstract

Proteolysis-targeting chimeras (PROTACs) represent a promising modality for targeted protein degradation, yet their structural complexity complicates systematic design and analysis. Bellerophon is a new computational tool that automatically decomposes PROTACs into their warhead, linker, and E3 ligase ligand directly from molecular structure. By enabling automated and standardized decomposition of degraders, the tool facilitates drug design at different levels: Bellerophon demonstrated versatility for moiety replacement (ARV-110), large-scale annotation (PROTAC-DB) and linker analysis (IRAK4 data set). The tool is freely available through a user-friendly web interface, with open-source code to encourage transparency and collaborative development in chemical biology and medicinal chemistry.

## Graphical abstract

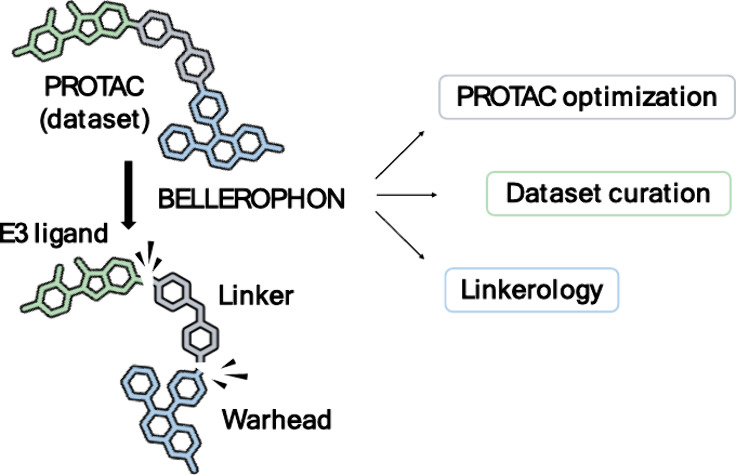

Targeted protein degradation (TPD) has emerged as a transformative strategy in medicinal chemistry, enabling the elimination of disease-driving proteins previously considered undruggable by traditional modulators (e.g., inhibitors, agonists, antagonists).^1–3^ TPD leverages cellular endogenous protein degradation machinery, primarily the ubiquitin-proteasome system (UPS), and lysosome-mediated pathways, to redirect selected proteins toward degradation.^2,4^ By eliminating rather than inhibiting disease-causing proteins, TPD technologies offer the potential for more complete and sustained therapeutic effects, and open new avenues for drug discovery against hard-to-target proteins.^5^

A wide range of TPD modalities have been developed, including lysosome-targeting chimeras (LYTACs), autophagy-targeting chimeras (AUTACs), molecular glues, and proteolysis-targeting chimeras (PROTACs).^6–9^ Among these, PROTACs are the most widely employed and clinically advanced, with about 50 candidates already in clinical trial.^5,10^ Examples include ARV-471, an oral estrogen receptor degrader currently in Phase III for breast cancer (NCT05654623, Figure 1A), and ARV-766, targeting the androgen receptor in prostate cancer (NCT05067140).^11,12^

**Figure 1:**
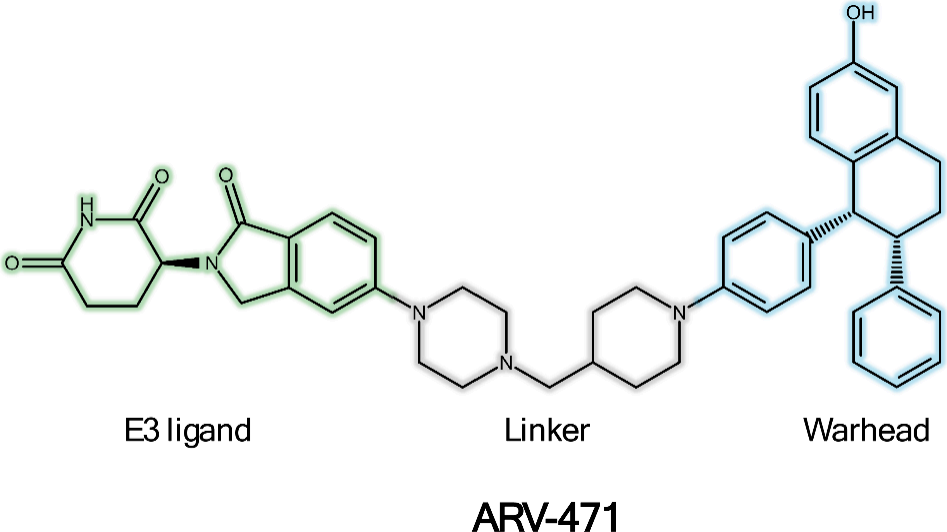
Structure of ARV-471, with the E3 ligand, linker, and warhead highlighted in light green, gray, and blue, respectively.

PROTACs are heterobifunctional molecules composed of three components: a ligand (or warhead) for the protein of interest (POI), an E3 ligase ligand that recruits the UPS machinery and a linker joining the two, as shown in Figure 1. By inducing proximity between the protein of interest with the E3 ligase, PROTACs promote ternary complex formation, ubiquitination, and subsequent proteasomal degradation of the POI.^13^

Heterobifunctional degraders have attracted tremendous interest in the drug discovery community for their unique ability to selectively degrade disease-related proteins that were previously considered undruggable.^13^ However, their large, flexible, and complex structures pose significant challenges for drug development, primarily due to poor solubility and limited permeability.^14,15^ Productive degradation requires several difficult-to-predict steps such as formation of a productive ternary complex and efficient ubiquitination.

To address these design challenges, several computational tools and curated databases have recently been developed to support the rational design of new PROTAC drugs, most of which are summarized in Table 1.^16^ In addition, several retrosynthetic planning platforms have been developed specifically for PROTAC synthesis (a detailed review of which lies beyond the scope of this paper).

**Table 1:** Main Tools Developed for PROTAC^a^

Tool name	Description	Use	ref
PROTAC-DB	Database	PD-PK data collection	^17^,^18^
PROTACpedia	Database	PD data collection	^19^
ELIOT	Online platform	E3 ligase navigator	^20^
E3 Ligase Landscape	Online platform	E3 ligase navigator	^21^
UbiHub	Online platform	Ubiquitination prediction	^22^
UbiBrowser	Online platform	Ubiquitination prediction	^23^
UbE3-APA	Python-based software	Ubiquitination prediction	^24^
PRosettaC	Web server	TC stability prediction	^25^
Method 1–5	MOE-based protocol	TC stability prediction	^26^,^27^
DegraderTCM	MOE-based protocol	TC stability prediction	^28^
Protac-Model	Protocol	TC stability prediction	^29^
AI-Based Tools			
DrugnomeAI	CNN-based framework	Target protaccability	^30^
MAPD	Random forest model	Target protaccability	^31^
E3 Binder Model	ML-classification based model	PROTAC design	^32^
DEVELOP	Python-based generative model	PROTAC design	^33^
LinkINVENT	Python-based generative model	PROTAC design	^34^
BOTCP	ML-based method (Bayesian optimization)	TC stability prediction	^35^
PRODE	Protocol	PROTAC design, TC stability prediction	^36^
Deep PROTAC	Deep learning classification model	Degradation prediction	^37^

a Abbreviations: PD-PK, pharmacodynamics-pharmacokinetics; TC, ternary complex; CNN, convolutional neural network; ML, machine learning.

In this context, the automated decomposition of PROTACs into their structural components, such as the two ligands and the linker, remains a highly nontrivial task, although significant progress has been made.^38^ Existing resources such as PROTAC-DB provide valuable information in this respect; however, they often rely on manually curated annotations that lack the flexibility for automated high-throughput workflows. Very recently, machine-learning (ML) frameworks like PROTAC-Splitter have been introduced to automate this process using Transformer and graph-based models trained on large synthetic data sets.^38^ While such ML approaches offer high-throughput potential, they can be susceptible to structural “hallucinations”, generating atoms not present in the parent molecule, and may show decreased accuracy when faced with structurally novel, out-of-distribution chemistry.^17,18^

Bellerophon (https://bellerophon-protac-decomposing-tool.streamlit.app/) addresses these challenges by offering a conceptually distinct, deterministic, and rule-based approach (Table S1). Our tool extracts the warhead, linker, and E3 ligand by applying consistent rules, standardizing the parsing of degrader structures, minimizing the risk of misclassification. By prioritizing chemical transparency, Bellerophon employs a bidirectional consistency check and strict structural filters. Rather than attempting to predict a decomposition pattern for every molecule, the tool only returns a result when the identified fragments account for the entire molecular composition of the parent PROTAC, ensuring atom conservation and chemical integrity in all successful outputs. Moreover, a high level of customizability allows users to easily expand or tailor reference libraries to navigate specific chemical spaces or proprietary data sets where ML models may lack sufficient training data. Such structure-driven decomposition facilitates not only the mining and comparison of existing degraders but also the *de novo* design of new ones, allowing chemists and modelers to reuse and recombine validated building blocks in novel configurations.

Bellerophon identifies the warhead, linker, and E3 ligand directly from the molecular structure of a PROTAC through a multistep procedure. Curated libraries of known warheads and E3 ligands were compiled from degraders reported in clinical trials and recent literature (for details refer to the Supporting Information). Moreover, to increase flexibility, the tool also allows users to upload custom libraries for PROTAC decomposition, ensuring compatibility with specific data sets or design objectives.

The algorithm first matches the input PROTAC against the warhead library; once a match is found, the corresponding fragment is removed, and the remaining portion is compared against the E3 ligand library to identify the linker (Figure 2A). The procedure is then repeated in reverse, starting from the E3 ligand, to ensure consistency. Only decompositions yielding identical linker fragments in both directions are retained (see the Supporting Information for details). To reduce the number of incorrect solutions, Bellerophon applies multiple structural filters, ensuring that the identified fragments account for the entire molecular composition of the PROTAC (Figure 2B; for details refer to the Supporting Information).

**Figure 2:**
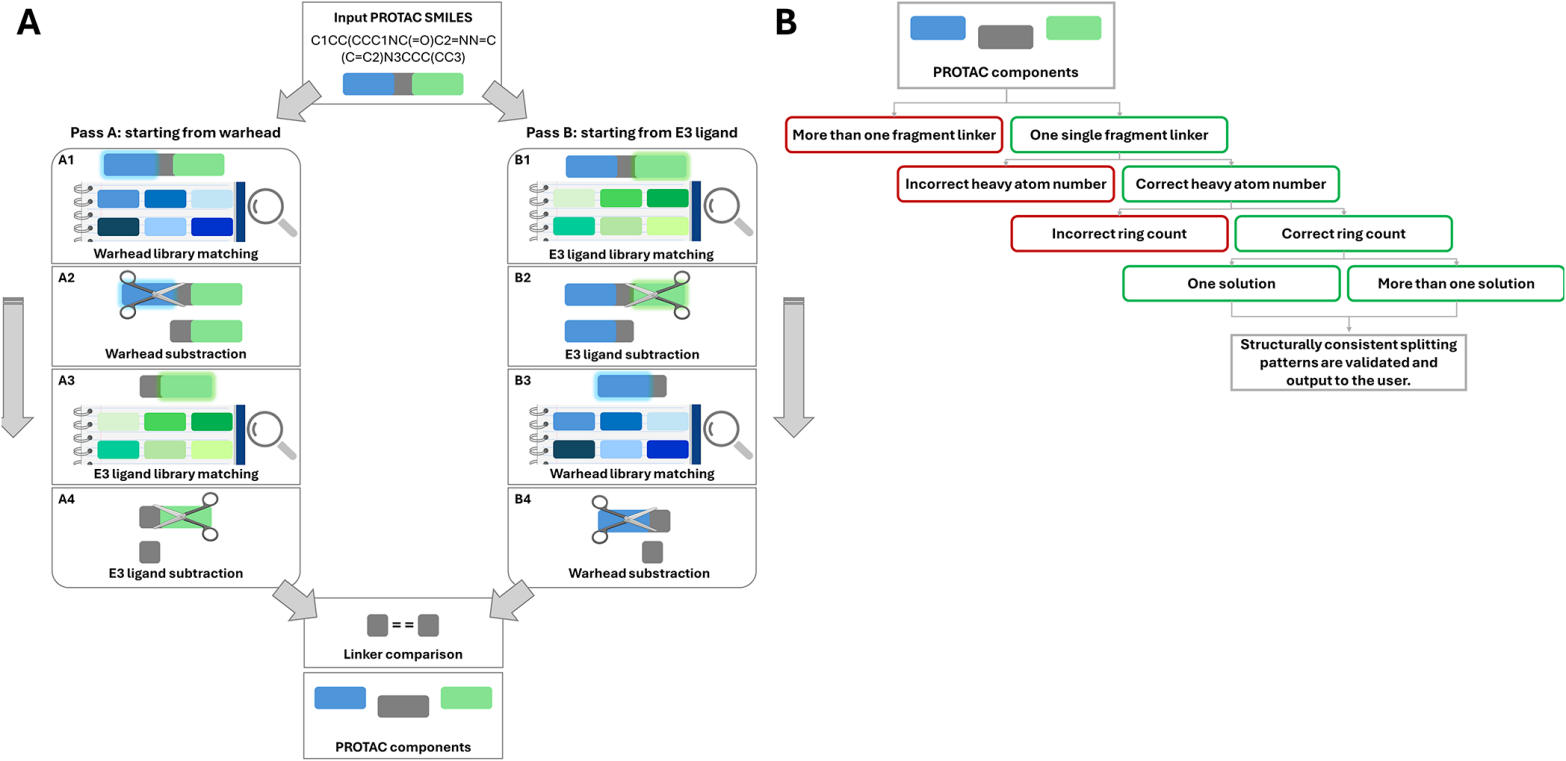
(A) Bellerophon function compares the PROTAC with a library of warheads and E3 ligands. When a match is found for the warhead, that portion is subtracted from the PROTAC, the remaining part (linker-E3 ligand) is compared to the list of E3 ligands. After the E3 ligand is matched and subtracted, the remaining portion is identified as the linker (pass A). The same procedure is performed starting from the E3 ligand (pass B); in the final step the linkers obtained are compared. (B) Filtering procedure to identify only correct solutions per PROTAC.

The tool was developed in Python using RDKit as the core cheminformatics toolkit and includes a web-based implementation (https://bellerophon-protac-decomposing-tool.streamlit.app/). Source code and curated input libraries are freely available on GitHub (https://github.com/giulia-apprato/Bellerophon/).

To ensure the chemical correctness of the decompositions from a medicinal chemistry perspective, Bellerophon was validated against a manually curated benchmark set of 49 commercially available PROTACs (validation set), including several currently in clinical trials including ARV-471, ARV766, and DT2216. The tool correctly decomposed all 49 compounds (100% success rate), with the resulting warheads, linkers, and E3 ligands matching the structural definitions reported in the literature (Table S2). For 23 of these PROTACs (47%), Bellerophon identified more than one consistent solution. In these instances, the tool reports all valid decompositions, leaving the final selection to the user based on their specific research or design goals. These alternative solutions typically represent different but plausible chemical boundaries, such as the inclusion or exclusion of a terminal amine or carbonyl group within the linker versus the ligand (Figure S1).

Bellerophon offers broad potential applications in drug discovery. Below three representative examples are reported: (i) the identification of alternative warheads for ARV-110, (ii) analysis of the PROTAC-DB data set, and (iii) linker analysis across a series of degraders targeting the IRAK4 protein.

As first application, we focused on ARV-110, one of the first PROTACs to enter clinical trials, developed to target the androgen receptor in metastatic castration-resistant prostate cancer (NCT03888612).^39^ The goal was to identify potential replacements for its warhead, a common need in PROTAC design. To this end, Bellerophon was used to obtain the SMILES string of ARV-110 warhead (Figure 3A), which was then employed to explore alternative AR ligands using ChemSpace Atlas (https://chematlas.chimie.unistra.fr/).^40^ This web server offers a user-friendly interface for chemical space navigation and prediction of biological activity. It includes approximately 40,000 generative topographic maps (GTMs) that accommodate over 500 million compounds from various chemical libraries (e.g., ZINC, https://zinc20.docking.org/ ^41^ and ChEMBL, https://www.ebi.ac.uk/chembl/ ^42^) spanning drug-like, fragment-like, and natural product-like spaces. ARV-110 warhead was projected using the SMILES string onto the AR protein (CHEMBL1871) specific activity map. Its location fell within a red region on the map, indicating a zone populated by compounds active toward the target (Figure 3B). Sixteen neighboring compounds with similar chemical features and predicted activity were identified as potential AR ligands. Figure 3C highlights representative candidates displaying favorable physicochemical properties and increased potential for replacing the original warhead.^43^

**Figure 3:**
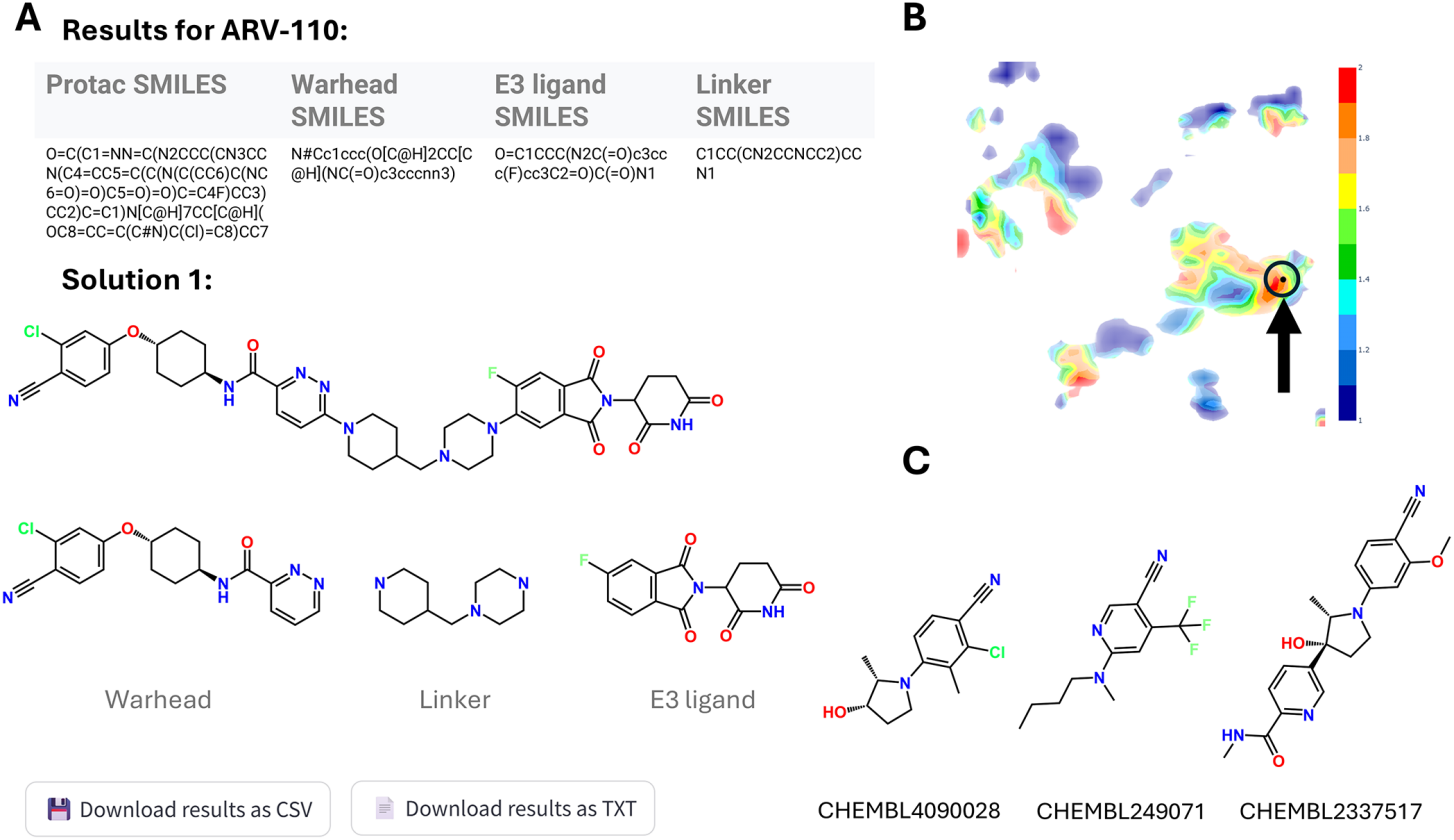
(A) ARV-110 decomposed by Bellerophon, with a table reporting respectively PROTAC, warhead, E3 ligand and linker SMILES, and the corresponding 2D structure of each moiety. (B) ChemSpace Atlas activity map for the androgen receptor (AR, CHEMBL1871), showing ARV-110 warhead (black dot) positioned within a red region of highly active compounds; blue regions indicate less active ones. (C) New candidate warhead structures identified through this analysis are shown.

This example demonstrates how Bellerophon, in combination with other tools (i.e., ChemSpace Atlas), can streamline early stage PROTAC design by enabling efficient and rational warhead replacement.

Another common need in drug discovery is the systematic analysis of PROTACs data sets. This type of analysis is particularly relevant in industrial settings, where degraders collections are often large and structurally heterogeneous. To assess Bellerophon’s capacity to handle such diversity, we evaluated its performance on PROTAC-DB (https://cadd.zju.edu.cn/protacdb/), the largest publicly available PROTAC database.

The effectiveness of this high-throughput analysis depends significantly on the quality of the reference libraries. The default Bellerophon libraries comprise 636 unique warheads and 105 E3 ligands curated from clinical and literature sources. To provide guidance on the chemical space covered, we analyzed their physicochemical properties (Figure S2 and the Supporting Information for 2D descriptor distributions). These libraries exhibit a broad distribution of size and polarity that aligns closely with FDA-approved drugs, with an average MW of 422–382 Da for Bellerophon warhead and E3 ligand moieties vs 333 Da for FDA drugs, suggesting the tool is well-suited for degraders built from established drug-like scaffolds.

Notably, the libraries also extend into higher molecular weight (up to ∼960 Da) and polarity (TPSA up to ∼274 Å^2^) regions, reflecting the unique structural requirements of TPD modalities.

Using these default curated libraries, we attempted to automatically decompose all PROTACs in the PROTAC-DB data set. A decomposition was considered correct when the three components (warhead, linker, and E3 ligand) matched the expected total number of heavy atoms and ring counts, and when the linker was returned as a single, continuous fragment. Under these criteria, Bellerophon correctly decomposed 51% of the PROTACs in PROTAC-DB (Figure 4A).

**Figure 4:**
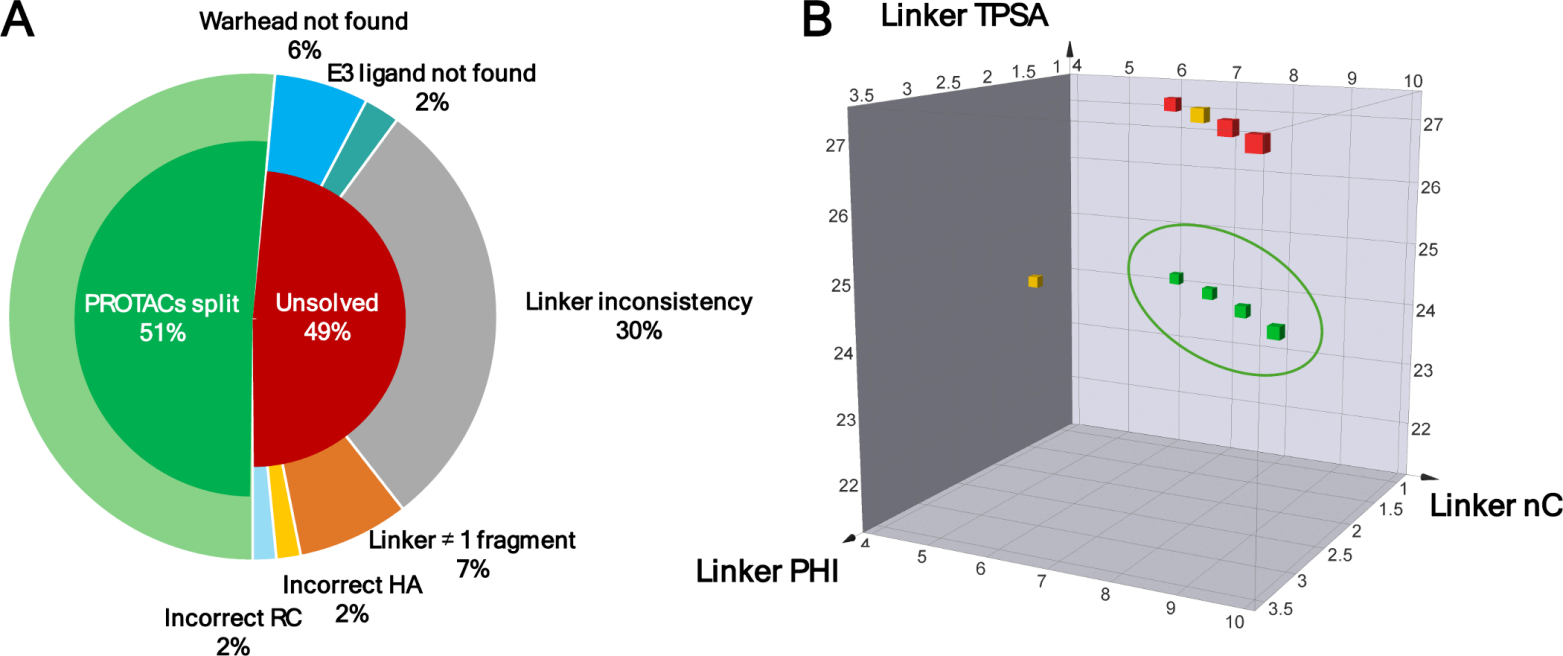
(A) Bellerophon performance on the PROTAC-DB data set. The chart shows the proportion of PROTACs correctly decomposed and those that could not be solved. Unsolved cases are further categorized by the main source of failure: missing warhead or E3 ligase in the reference libraries, linker inconsistency between pass A and B, multiple linker fragments, and discrepancies in heavy atom or ring counts. (B) IRAK4 targeting PROTACs analyzed in terms of number of carbon atoms (nC), Kier’s flexibility index (PHI), and topological polar surface area (TPSA). Degraders are colored according to the degradation efficacy: strong degraders are shown in green, moderate in yellow and poor degraders in red.

In several cases, multiple valid decomposition patterns were identified, typically arising from alternative E3 ligand matches or boundary overlaps (Figure S1). To manage such results and ensure reproducibility, we recommend tailoring the reference libraries to the specific research objective.

- Targeted Data sets: for high-precision analysis of known compounds, removing smaller or redundant warhead fragments minimizes overlapping matches and simplifies the output.
- Large-Scale Mining: conversely, for heterogeneous data sets like PROTAC-DB, a more diverse range of fragments, including smaller overlapping substructures, increases the probability of successfully decomposing a wider variety of chemotypes.

Among the remaining 49% of cases, the most frequent source of failure (≈30%) was inconsistency between the two linker fragments generated in pass A and B (Figure 2A). Additional failures stemmed from unmatched moieties in the reference libraries (warhead and E3 ligase matching failed in ≈6% and 2% of cases, respectively) and from incomplete substructure matches that produced multiple disconnected linker fragments (≈7%). For the convenience of the user, all unresolved cases are exported to a separate file, allowing for targeted library expansion. Notably, the tool’s performance is directly responsive to such updates: we observed that just adding ten additional warheads to the library increased the success rate by 4%, mainly by reducing linker and ring-count inconsistencies (not shown). This demonstrates that Bellerophon’s architecture is not only robust for standard degraders but is also easily adaptable to novel E3 ligases and unconventional bifunctional molecules.

Overall, Bellerophon demonstrated a quite robust and scalable performance on a large and chemically diverse data set. Its accuracy is strongly influenced by the completeness and quality of the input reference libraries, while failed or ambiguous cases reflect the intrinsic structural complexity of PROTACs and the challenges of automating moiety-level recognition.

As a final example, we demonstrate how Bellerophon can be applied to investigate the influence of linker properties on degradation efficiency, highlighting its potential for structure–activity relationship studies in PROTAC drug discovery. This was exemplified through the analysis of degraders targeting interleukin-1 receptor-associated kinase 4 (IRAK4), a mediator of inflammatory signaling involved in autoimmune disorders. For this application, we selected a data set of IRAK4 degraders inspired by KT-474, the first IRAK4-targeting PROTAC to progress to phase II clinical trials for atopic dermatitis (NCT04772885). Liu and colleagues reported a series of 25 degraders developed to improve the metabolic stability and pharmacokinetic behavior of the DE5 lead compound through linker rigidification strategies.^44^ While this sample size is small, the data set is well suited for assessing linker’s effects, as all degraders share the same DE5-derived warhead and thalidomide E3 ligand, differing only in linker composition and attachment point (either the 4′ or 5′ position on the thalidomide scaffold).

Bellerophon was used to decompose each PROTAC and isolate the linker. Physicochemical descriptors were then computed, including 2D descriptors related to size (molecular weight, MW), flexibility (Kier’s flexibility index), hydrophobicity (number of carbons, nC), and polarity (topological polar surface area, TPSA; hydrogen bond donors, HBD; and acceptors, HBA). The compounds were grouped by degradation efficiency into poor, moderate, and strong degraders (refer to the Supporting Information for details).

Analysis of the resulting data set revealed that PROTACs with linker attachment at the 4′ position generally showed lower degradation efficiency (not shown), whereas those linked at the 5′ position (degraders shown in Figure 4B) included both active and less active examples. To exclude the effect of the attachment point, only 5′-linked degraders were further analyzed. In Figure 4B, these degraders are plotted according to linker polarity (TPSA), flexibility (PHI), and length (number of carbon atoms). Highly active compounds clustered in a distinct region, featuring linkers that matched fewer active degraders in length and flexibility, yet exhibited markedly lower polarity. This observation seems to suggest that, for linkers of comparable size and conformational freedom, reduced polarity favors target degradation. However, it must be emphasized that because the data set is limited, these findings are correlative rather than causal and this should be viewed as illustrative example of how Bellerophon can facilitate linkerology studies by enabling rapid extraction and analysis of linker substructures.

Overall, Bellerophon offers a quite robust, fully automated approach for decomposing heterobifunctional degraders directly from their SMILES code. Unlike existing resources, it employs a deterministic, rule-based algorithm to identify and extract the actual molecular subcomponents. Through three representative applications, we demonstrated Bellerophon’s versatility and potential impact in drug discovery. Its customizable framework is particularly suited for analyzing proprietary data sets, allowing users to upload tailored warhead and E3 ligand libraries and thus adapt the workflow to specific chemical spaces.

However, the primary limitation of the current approach is its reliance on manually curated reference libraries for warheads and E3 ligands. While these libraries provide the transparency and deterministic control necessary for high-precision medicinal chemistry, they require periodic updates to keep pace with the rapid evolution of TPD chemotypes. Future developments integrating AI-based recognition of warheads and E3 ligands could potentially resolve this dependency, enabling the tool to identify novel moieties autonomously.

Despite this current limitation, the integration of Bellerophon into virtual screening pipelines already enables rapid annotation and sorting of large PROTAC collections, facilitating scaffold-hopping and design optimization. Finally, the same principle of molecular decomposition can be extended to other heterobifunctional modalities, such as LYTACs and AUTACs, underscoring Bellerophon’s potential as a versatile tool for the rational exploration of targeted protein degradation chemistry.

## Supplementary Material



## Data Availability

PROTACs validation data set, IRAK4 PROTACs SMILES and calculated properties are available. Bellerophon is available on our site as well: https://www.medchembeyondlab.unito.it/our-tools.

## Notes

Supporting Information is available free of charge on the ACS Publications Web site. It includes: Libraries preparation, Bellerophon workflow, Implementation, GUI interface, and Application details regarding PROTAC-DB and IRAK4 degraders (PDF)

## Abbreviations

TPD: targeted protein degradation
UPS: ubiquitin-proteasome system
LYTACs: lysosome-targeting chimeras
AUTACs: autophagy-targeting chimeras
PROTACs: proteolysis-targeting chimeras
POI: protein of interest
MW: molecular weight
PHI: Kier’s flexibility index
nC: number of carbon atoms
TPSA: topological polar surface area
HBD: hydrogen bond donors
HBA: hydrogen bond acceptors
CRBN: cereblon
IRAK4: interleukin-1 receptor-associated kinase 4
