# Supplementary material for: Bellerophon: An Automated Tool for PROTAC Decomposition
Source: ACS Med Chem Lett. 2026 Apr 10;17(5):1043–9. doi: 10.1021/acsmedchemlett.5c00769 (PMC13181455; doi:10.1021/acsmedchemlett.5c00769)
Supplement: Supplementary file 1 [file ml5c00769_si_001.pdf]

Supporting Information: Bellerophon: an Automated Tool for PROTAC Decomposition

Giulia Apprato[1*], Matteo Bertola[2], Amelia Locatelli[1], Giulia Caron[1*], Andrea Mauri[2], Giuseppe Ermondi[1*]

[1] Department of Molecular Biotechnology and Health Sciences, University of Turin, Torino, 10126, Italy

[2] Alvascience Srl, Lecco, 23900, Italy

Libraries preparation

The initial step involved the manual curation of the warhead and E3 ligand libraries. PROTACs currently in clinical trials and reported in the literature were included. The dataset is available on github. Molecules were processed using AlvaMolecule (v2.0.6, Alvascience, www.alvascience.com) for standardization and duplicate removal. [43] The curated compound libraries were then imported into Python (http://www.python.org, vv. 3.10.12) and further processed using RDKit open-source cheminformatics library (https://www.rdkit.org/, vv. 2025.09.1).

Bellerophon workflow

The process begins by comparing the input PROTAC structure with a curated dictionary of known warheads (Figure 2A, step A1). If a match is found, the warhead is subtracted from the full structure (step A2), and the remaining portion, composed of the linker and E3 ligand, is compared against a second dictionary of E3 ligands (step A3). Once a match is identified, the E3 ligand is removed (step A4), and the remaining structure is assigned as the linker.

To ensure robustness, the same procedure is repeated starting from the E3 ligand instead of the warhead (Figure 2A, pass B, steps B1–B4). The two resulting linker fragments, obtained from the two independent passes, are then compared. If the linker fragments are not identical, the decomposition is discarded, as inconsistency indicates a likely partial or incorrect match of the warhead/E3 ligand moieties.

To further reduce false positives caused by partial substructure matches, several filtering steps are applied (Figure 2B). First, only linkers that consist of a single, continuous molecular fragment are accepted. Next, the number of heavy atoms and the number of rings (aromatic and aliphatic) are calculated for the entire PROTAC and for each of the three identified fragments. The properties of the fragments must sum exactly to those of the full molecule; otherwise, the decomposition is rejected. For the same compound several solutions may be available; in that case it is up to the user to choose the preferred solution.

Implementation

Bellerophon was developed in Python, using several key libraries. RDKit served as the core cheminformatics toolkit used for molecular manipulation, descriptor calculation, and substructure matching. Pandas and Numpy were used respectively for data handling and numerical operations.

The process begins with the import of curated libraries of warheads and E3 ligands. By default, these libraries are prepared and curated by us and include the most recent PROTACs in clinical trials as well as additional degraders manually collected from multiple sources, including PROTAC-specific databases. However, users can also provide their own custom dictionaries of warheads and E3 ligands in .sdf format, allowing flexibility and expansion beyond the default set. The user can either paste a list of PROTACs (name and SMILES) or upload a .txt, .csv, or .sdf file containing this information. Each SMILES is then automatically converted into an RDKit molecular object (Mol), after which the decomposition procedure is initiated. If any SMILES string is invalid, the tool produces an error message.

Fragment removal is achieved using RDKit's DeleteSubstructs function. The tool then calculates various molecular descriptors using rdMolDescriptors, including the number of heavy atoms and the number of rings.

The full Bellerophon script is available on GitHub (https://github.com/giulia-apprato/Bellerophon/) and can be easily adapted or integrated into broader computational pipelines for heterobifunctional degrader design. The dictionaries used in this study are also included in the repository.

GUI interface

The graphical interface was implemented using Streamlit (https://streamlit.io/), an open-source Python framework that allows for rapid development of interactive web applications. The user interface was designed with the objective of enabling users to navigate the system with minimal guidance and with few expertise in the computational field.

Comparison between PROTAC-splitter and Bellerophon

Table S1: Bellerophon-PROTAC-splitter comparison.

Feature	Bellerophon	PROTAC-splitter
**Logic**	Deterministic, rule based.	Probabilistic, learned from training data.
**Atom conservation**	Guaranteed by structural filters; fails if inconsistent.	Risk of extra or missing atoms (“hallucinations”).
**Customization**	Immediate through user-editable SMILES libraries.	Complex, it requires model retraining or fine-tuning.
**Transparency**	High; every split is traceable to a library. entry	Low; "black-box" predictions.
**Main limitation**	Accuracy is sensitive to the quality and completeness of user-curated reference libraries; not all PROTACs are provided with a solution.	Performance drops significantly on out-of-distribution (OOD) data; risk of invalid SMILES.

Manual validation and multiple solutions analysis

To ensure the chemical correctness of the rule-based decomposition from a medicinal chemistry perspective, a benchmark dataset of 49 commercially available PROTACs was manually curated. This set includes several degraders currently in Phase I–III clinical trials (e.g., ARV-471, ARV-110, KT-474) to test the tool against therapeutically relevant and structurally complex chemotypes. Table S1 shows the validation dataset. For each PROTAC, the name, SMILES, target, E3 ligase, Disease area, Correct solution number among the ones provided by Bellerophon, Warhead, E3 ligand and linker SMILES and the main differences between different solutions for the same compound (for the complete version showing PROTAC, warhead, E3 ligand and linker SMILES refer to excel file).

As reported in the table and in the excel file, common variations between decomposition solutions for the same PROTAC include:

- Functional group partitioning: inclusion or exclusion of terminal atoms (e.g., a carbonyl oxygen or an amine nitrogen) within the E3 ligand versus the linker or the warhead versus the linker.
- Linker length and boundary definitions: differences in how alkyl chains are partitioned when substructure matches overlap between the reference libraries and the PROTAC scaffold.

In these instances, Bellerophon provides all valid interpretations. It is the responsibility of the user to select the solution that best aligns with their specific synthetic precursors, metabolic stability considerations, or structure–activity relationship (SAR) objectives (Fig. S2).

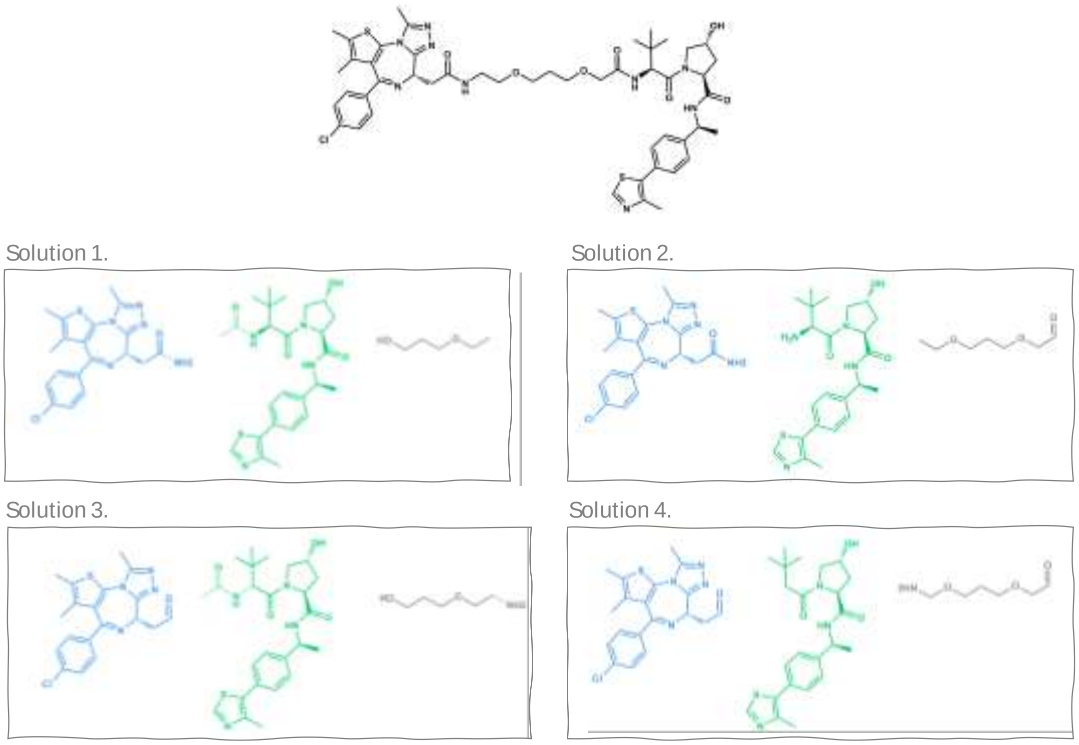

***Figure S1**: Structural decomposition patterns identified by Bellerophon for ARV-771. The four solutions represent alternative partitioning of the "borderline" atoms between the linker and the functional moieties. Solution 2 (the literature-reported definition) assigns the amine nitrogen to the warhead and the carbonyl oxygen to the linker. In contrast, Solution 3 assigns the amine nitrogen to the linker and the carbonyl oxygen to the E3 ligand, while Solution 4 assigns both atoms to the linker.*

Table S2: Summary of validation dataset (49 PROTACs) respectively with PROTAC name, target and E3 ligase recruited, disease area of competence, number of solutions identified by the tool and differences among these solutions

Number	Name	Target	E3 Ligase	Disease Area	Solutions	Differences
**1**	ACBI1	SMARCA2/4	VHL	SMARCB1-deficient Cancers	1	-
**2**	ACBI2	SMARCA2	VHL	Lung Cancer	1	-
**3**	ARCC-4	AR	VHL	Prostate Cancer (Enzalutamide-resistant)	1	-
**4**	ARD-69	AR	VHL	Castration-Resistant Prostate Cancer	1	warhead stereochemistry
**5**	ARV-110	AR	CRBN	Prostate Cancer	1	-
**6**	ARV-393	BCL6	CRBN	Advanced non-Hodgkin lymphoma	1	-
**7**	ARV-471	ER	CRBN	Breast Cancer	1	-
**8**	ARV-766	AR	CRBN	Castration-Resistant Prostate Cancer	1	-
**9**	ARV-771	BET	VHL	Castration-Resistant Prostate Cancer	2	Linker length
**10**	ARV-825	BRD4	CRBN	Lymphoma/Burkitt's	4	Amine in the linker/warhead/ E3
**11**	BETD-246	BET	CRBN	Triple-Negative Breast Cancer	2	Amine in the linker/E3
**12**	BGB-16673	BTK	CRBN	B-cell Malignancies	1	-

Number	Name	Target	E3 Ligase	Disease Area	Solutions	Differences
**13**	BMS-986365	AR	CRBN	metastatic castration-resistant prostate cancer	1	-
**14**	BMS-986458	BCL6	CRBN	B-cell non-Hodgkin's lymphoma	1	-
**15**	BSJ-03-123	CDK6	CRBN	Leukemia	1	-
**16**	BSJ-4-116	CDK12	CRBN	Advanced Solid Tumors	1	-
**17**	CFT1946	BRAF (V600E)	CRBN	Cancer research	1	-
**18**	CFT8634	BRD9	CRBN	Synovial Sarcoma	1	-
**19**	dBET1	BRD4	CRBN	Hematologic Cancers	2	Amine in the linker/warhead
**20**	dBET6	BRD4	CRBN	Acute Myeloid Leukemia	2	Amine in the linker/warhead
**21**	dCBP-1	CBP/p300	CRBN	Multiple Myeloma	1	Amine in the linker/E3
**22**	DT2216	BCL-XL	VHL	T-cell Leukemia / Small Cell Lung	2	Linker length
**23**	FHD-609	BRD9	CRBN	adrenocortical carcinoma	1	-
**24**	FMF-06-098	multiple-target kinase	CRBN	Hematologic Cancers	2	Linker length
**25**	GNE-987	BRD4	VHL	Advanced Solid Tumors	1	-
**26**	INY-03-041	pan-AKT	CRBN	Cancer research	1	-
**27**	KT-253	MDM2	CRBN	hematologic and solid tumors	1	Linker length
**28**	KT-413	IRAK4	CRBN	Cancer research	2	Amine in the linker/E3
**29**	LC-2	KRAS G12C	VHL	Non-Small Cell Lung Cancer	1	-
**30**	MD-224	MDM2	CRBN	Leukemia / Solid Tumors	1	Linker length
**31**	MG-277	GSPT1	CRBN	Acute Myeloid Leukemia	1	-
**32**	MS154	EGFR	VHL	Lung cancer	1	-
**33**	MS432	MEK1/2	VHL	KRAS-mutant Cancers	2	Carbonyl in the linker/E3
**34**	MS67	WDR5	VHL	MLL-rearranged Leukemia	2	Carbonyl in the linker/E3
**35**	MT-802	BTK	CRBN	B-cell Lymphoma	1	Amine in the linker/E3
**36**	MTX-23	AR	VHL	Metastatic Prostate Cancer	2	Linker length
**37**	MZ1	BRD4	VHL	Solid Tumors	1	Amine in the linker/warhead
**38**	NX-2127	BTK, IKZF1/3	CRBN	Chronic Lymphocytic Leukemia	1	-
**39**	NX-5948	BTK	CRBN	Lymphoma / Rheumatoid Arthritis	1	-
**40**	PRT3789	SMARCA2	VHL	Non-Small Cell Lung Cancer	1	Linker length
**41**	QCA570	BRD4	CRBN	Advanced Solid Tumors	1	-
**42**	SD-36	STAT3	CRBN	Leukemia / Lymphoma	1	-
**43**	SIAIS178	BCR-ABL	VHL	Chronic Myeloid Leukemi	1	-
**44**	SJF620	BTK	CRBN	Lymphoma	1	-
**45**	SNS-032-T	CDK9	CRBN	Acute Myeloid Leukemia	2	Amine in the linker/E3

**46**	TL12-186	multiple-target kinase	CRBN	Cancer research	4	Linker length and amine in the linker/E3
**47**	TMX-4153	PIP4K2C	VHL	immune modulation-related diseases	2	Linker length
**48**	XZ739	BCL-XL	CRBN	Leukemia / Senolytic	1	Amine in the linker/E3
**49**	ZXH-3-26	BRD4	VHL	Hematologic Malignancies	2	Amine in the linker/E3

PROTAC-DB application

PROTAC-DB was downloaded on 01.10.2025. The dataset was curated using AlvaMolecule (v2.0.6, Alvascience, www.alvascience.com) for standardization and duplicate removal.[43] Overall, 6109 unique degraders were identified.

Default libraries description

A

Property	Validation dataset	Warhead	E3 ligand	FDA approved
MW min	192.1	58.0	178.1	22.9
MW max	917.2	960.7	679.4	984.4
MW average	426.1	422.2	382.3	333.5
TPSA min	17.1	12.5	30.2	3.2
TPSA max	241.2	274.5	197.5	392.8
TPSA average	99.6	104.2	105.2	88.9
PHI min	2.0	1.5	1.7	0.0
PHI max	11.8	15.4	8.2	479.3
PHI average	4.6	5.0	4.6	5.4

B

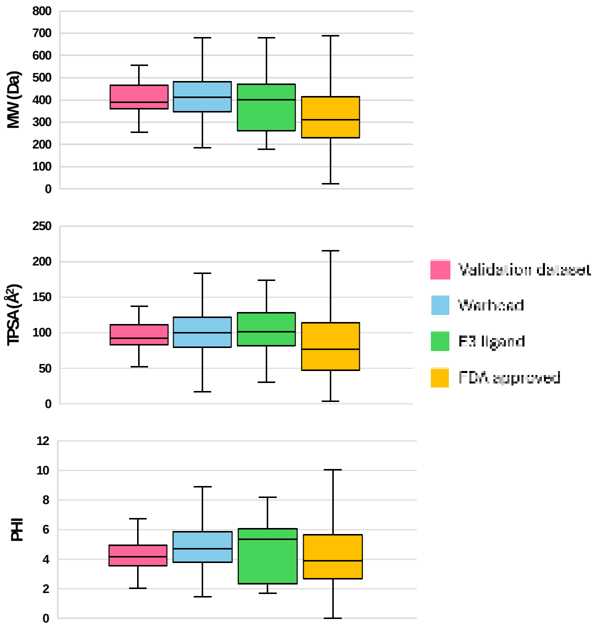

C

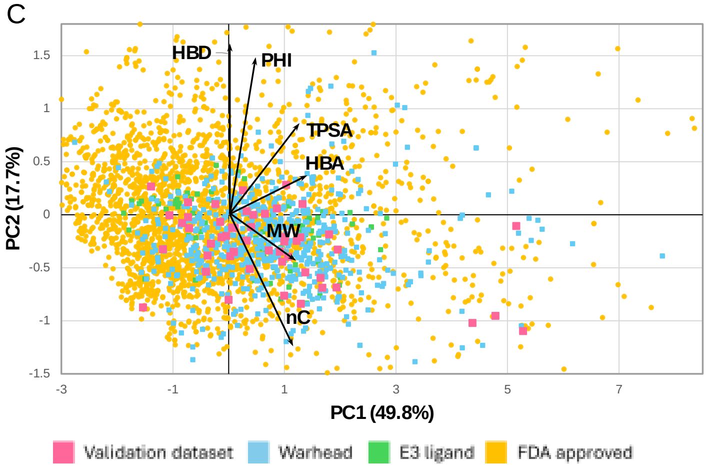

Figure S2. *Bellerophon default libraries characterization. (A) Summary statistics (minimum, maximum, and average values) for molecular weight (MW), topological polar surface area (TPSA), and Kier's flexibility index (PHI) across the warhead and E3 ligand libraries, the validation dataset (n=49), and a reference set of FDA-approved drugs available on DrugBank (https://go.drugbank.com/). (B) Box plots showing the distribution of size (MW), polarity (TPSA) and flexibility (PHI) across the datasets, highlighting the structural diversity and heterogeneity of the default reference libraries. (C) PCA scores and loading plot, illustrating the high degree of overlap between the Bellerophon libraries and the space occupied by approved drugs.*

IRAK4 degraders application

Since DC_{50} and D_{max} values were not available for the entire dataset, degradation percentages at 5 uM and 0.5 uM were used instead. An average degradation value was calculated and employed for classification purposes. IRAK4 degraders were categorized as strong (average degradation > 70%), moderate (50-70%), or poor (<50%).

Each degrader was processed with Bellerophon to isolate the linker moiety, for which physicochemical descriptors were calculated using AlvaDesc[43] (v2.0.6, Alvascience, www.alvascience.com). DataWarrior was then used to visually inspect the two compound series differing only in the linker attachment point on the thalidomide ring, and to explore potential correlations between degradation potency and physicochemical properties.
